# Commentary on: Aesthetic Values of the Buccal Fat Pad Excision in Middle-Aged Patients

**DOI:** 10.1093/asjof/ojac027

**Published:** 2022-05-02

**Authors:** James M Stuzin

**Affiliations:** University of Miami School of Medicine, Miami, FL, USA

See the Original Article here.

This article^1^ reviews the indications, technical options, and aesthetic improvement associated with buccal fat removal in Asian patients. The aesthetic indications for this procedure are specifically in the full face patient, providing for a reduction in submalar volume, producing a more tapered angular facial shape following fat removal. This invited video commentary discusses both the indications and limitations of buccal fat removal (Video). Caution of the overuse of buccal fat removal in the older patient is discussed, where buccal fat may accentuate submalar deflation, which is common in this age group. The limitations of buccal fat removal in improving the appearance of the lower cheek and jowl are emphasized.
